# Variational quantum algorithm for node embedding

**DOI:** 10.1016/j.fmre.2023.10.001

**Published:** 2023-10-14

**Authors:** Zeng-rong Zhou, Hang Li, Gui-Lu Long

**Affiliations:** a Research Center for Quantum Sensing, Zhejiang Lab, Hangzhou 311121, China; b Beijing Academy of Quantum Information Sciences, Beijing 100193, China; c State Key Laboratory of Low-Dimensional Quantum Physics and Department of Physics, Tsinghua University, Beijing 100084, China

**Keywords:** Quantum machine learning, Quantum computation, Node embedding, Variational quantum algorithm, Nuclear magnetic resonance

## Abstract

Quantum machine learning has made remarkable progress in many important tasks. However, the gate complexity of the initial state preparation is seldom considered in lots of quantum machine learning algorithms, making them non-end-to-end. Herein, we propose a quantum algorithm for the node embedding problem that maps a node graph’s topological structure to embedding vectors. The resulting quantum embedding state can be used as an input for other quantum machine learning algorithms. With O(log(N)) qubits to store the information of N nodes, our algorithm will not lose quantum advantage for the subsequent quantum information processing. Moreover, owing to the use of a parameterized quantum circuit with O(poly(log(N))) depth, the resulting state can serve as an efficient quantum database. In addition, we explored the measurement complexity of the quantum node embedding algorithm, which is the main issue in training parameters, and extended the algorithm to capture high-order neighborhood information between nodes. Finally, we experimentally demonstrated our algorithm on an nuclear magnetic resonance quantum processor to solve a graph model.

## Graphical abstract

**Figure:**
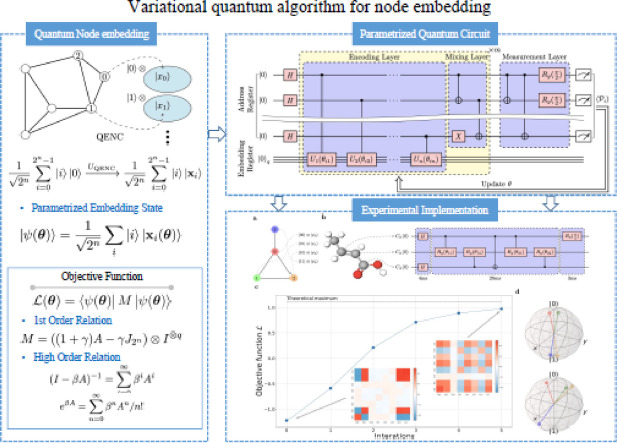

## 1 Introduction

Quantum computing has made significant progress in the field of machine learning. Quantum machine learning (QML), which completes the machine-learning tasks using quantum computers, has proven to have great potential [1], [2], [3]. QML algorithms take advantage of quantum superposition and parallelism for processing large-scale data. However, many existing QML algorithms [4], [5], [6] assume that there is already a quantum database that can be prepared quickly [7], [8]. Recently, some authors have pointed out that the quantum advantage of these algorithms may arise from this assumption rather than from the algorithms themselves [9]. Rapid preparation of the input state has now become a critical issue in the design of QML algorithms.

Machine learning, which constructs models by learning from data, is currently a central part of artificial intelligence. Besides its innovative approach to model training, feature engineering, which extracts and selects the most relevant features from raw data to create a predictive model, is a crucial step in machine learning [10], [11]. Good data characteristics for a machine learning task always provide a higher upper limit of machine learning model performance. However, we cannot precisely determine good features for some types of data, such as words and knowledge, simply through manual analysis. We do know the exact relations among these data, which can form a graph. Thus, a good solution for feature extraction is to learn the representation of the elements in a graph [12], [13]. Specifically, we aim to present a feature vector for each data point based on a graph using a learning model called node embedding. Node embedding is defined as encoding nodes into a latent space in which the geometric relations reveal the topological structure of the original graph [14]. Many node embedding methods have been proposed in recent decades, and they can be divided into three categories: factorization-based [15], [16], [17], random walk-based [18], [19] and deep neural network-based [20], [21], [22]. Node embedding is now an essential step in machine learning tasks on graphs, which play a crucial role in modern life. Some critical applications involving graph models are recommendation systems based on social networks [23], and drug side-effect prediction [24], among others [25]. However, when the scale of the input graph and dimension of the feature vector are large, the training process of these algorithms consumes considerable computing resources. To address these issues, assistance from quantum computing is required.

In this study, we propose and demonstrate a quantum node embedding (QNE) algorithm on a nuclear magnetic resonance (NMR) quantum computing platform. Using classical graph information as input, we can produce a quantum node embedding vector for each node in a graph. The embedding algorithm produces a quantum state containing the embedding and address states of each node. Similar to the classical case in which the embedding vectors can be used as feature vectors for subsequent machine learning tasks, the resulting quantum state can also be used as input for other QML algorithms. In the QNE algorithm, we use a parameterized quantum circuit (PQC) [26], [27] to generate quantum feature vectors. After training, the embedding state generated by the classical parameters can be used as an efficient quantum database for QML algorithms, making them end-to-end programs. A previous study built a similar framework to ours [28]. However, their algorithm for constructing the quantum embedding of a graph is based on an approximate approach, which can only achieve a linear speedup. For some embedding graphs, our algorithm can be proven to have quantum advantages, which is essential for pratical quantum algorithms. Furthermore, our algorithm was extended to capture higher-order neighborhood relations between nodes in a graph without losing quantum advantages.

## 2 Theory and algorithm

**Fig. 1:**
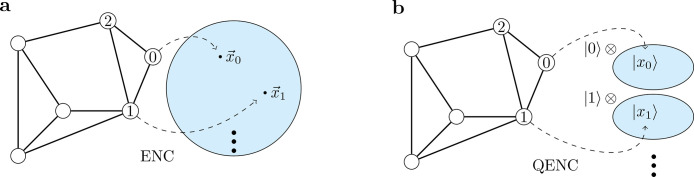
**Node embedding in the classical and quantum cases.** (a) In the classical case, node embedding maps the topological relation of nodes to the vectors in a multidimensional Euclidean space. (b) In the quantum case, node embedding maps the same graph to an entangled quantum state. All nodes in the graph are encoded in states in a Hilbert subspace, tensored with label states that are uniformly superposed in the label space.

In a classical node embedding task, an embedding vector $x_{i}\in R^{d}$ for every node $v_{i}$ in a graph is provided, as shown in Fig. 1a. The encoder-decoder framework proposed by Hamilton et al. [12] defines an encoder function as $\text{ENC}:V\to R^{d}$, where V contains all vertices in a graph G, and d is the dimension of the embedding Euclidean space. In our QNE algorithm, the quantum encoder $U_{\text{QENC}}$ is defined to provide quantum state vectors $|x_{i}\rangle$ in a Hilbert space. In Fig. 1b, each node in the original graph is encoded as a state vector $|x_{i}\rangle$ in the Hilbert subspace and labeled by an orthogonal basis vector |i〉. Specifically, we want to construct $U_{\text{QENC}}$ to realize the following:

(1) $$\frac{1}{\sqrt{2^{n}}}\sum_{i=0}^{2^{n}-1}|i\rangle |0\rangle \overset{U_{\text{QENC}}}{\to }\frac{1}{\sqrt{2^{n}}}\sum_{i=0}^{2^{n}-1}\left|i\rangle \right|x_{i}\rangle ,$$

where the resulting quantum state serves as the input state for subsequent QML tasks, such as quantum clustering and classification.

In the classical case, we define a decoder function $\text{DEC}:R^{d}\times R^{d}\to R^{+}$ to reconstruct the graph statistics from the node’s embedding vectors, which ENC generates. This function builds a measure among embedding vectors in $R^{d}$, which reveals their relations. For example, we can select the decoder function as $\text{DEC}\left(x_{i},x_{j}\right)=x_{i}^{T}x_{j}$. In the original graph, we can then also construct a function $S(v_{i},v_{j})$ to measure the similarity between nodes. In a simple case, we can choose $S(v_{i},v_{j})$ as $A_{ij}$, which is the corresponding element of the adjacency matrix A. For an undirected and weightless graph G, we define its adjacency matrix A, where $A_{ij}=1$ if and only if there is an edge between nodes i and j, and $A_{ij}=0$ otherwise. Our goal is for the geometry in the embedding space to reveal the node similarity in the graph. We define an objective function as:

(2) $$L=\sum_{i,j}\ell \left[\text{DEC}\left(x_{i},x_{j}\right),S\left(v_{i},v_{j}\right)\right]$$

to minimize their difference through optimization. In the classical case, the objective function can be the mean square or cross-entropy loss.

For ease of quantum information processing, we define the objective function as:

(3) $$L=\sum_{i,j}A_{ij}\langle x_{i}|x_{j}\rangle -\gamma \left(1-A_{ij}\right)\langle x_{i}|x_{j}\rangle$$

The first term in the function requires the inner product between the embedded vectors of two connected vertices in the graph to be large. The second term can be viewed as a penalty for reducing the inner product between the embedded vectors of the two unconnected vertices in the graph. γ is a hyperparameter for balancing these two terms. Therefore, the larger the value of the objective function, the closer the geometric relation among embedding vectors is to the structure of the graph. $U_{\text{QENC}}$ is realized through a PQC, which produces a quantum state characterized by a parameter vector θ:

(4) $$|\psi (\theta )\rangle =\frac{1}{\sqrt{2^{n}}}\sum_{i}\left|i\rangle \right|x_{i}\left(\theta \right)\rangle$$

Expressing the adjacency matrix in the computational basis as $A=\sum_{i,j}A_{ij}|i\rangle \langle j|$, we have $\langle \psi |A⊗I^{⊗q}|\psi \rangle =\sum_{i,j}A_{ij}\langle x_{i}\left(\theta \right)|x_{j}\left(\theta \right)\rangle$. Then the objective function becomes L(θ)=〈ψ(θ)|M|ψ(θ)〉 where:

(5) $$M=\left(\left(1+\gamma \right)A-\gamma J_{2^{n}}\right)⊗I^{⊗q}$$

$J_{2^{n}}$ denotes an all-ones matrix, which can be decomposed as ${\left(I+X\right)}^{⊗n}$. X and I denote the Pauli-X matrix and identity operator, respectively. Finally, the QNE algorithm is constructed to optimize the parameters in $U_{\text{QENC}}$ to maximize the value L(θ), which can be estimated from measurement results of the quantum circuit. The implementation detailes of the algorithm are provided in Supplementary Section 1.

**Fig. 2:**
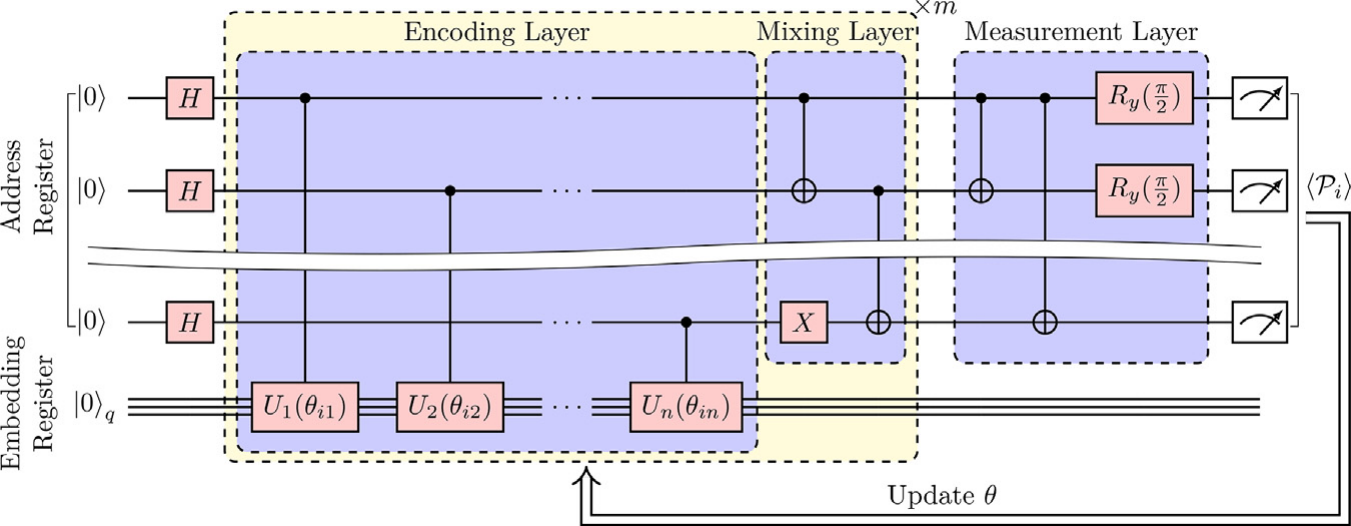
**Quantum circuit for our quantum node embedding algorithm.** The quantum circuit mainly consists of three parts: (i) Hadamard gates applied to each qubit of the address register, preparing a uniform superposition. (ii) A PQC with encoding and mixing layers cycling alternately. (iii) Measurement layer for measuring the expectation of Pauli product series.

According to Eq. 1, the quantum encoder circuit will produce quantum embedding states in a Hilbert subspace, labeled by vertex indices. With the assumption that those $\left|i\rangle \right|x_{i}\left(\theta \right)\rangle$ states are uniformly superposed, we propose a PQC ansatz for this problem, as shown in Fig. 2. The qubits in the circuit are divided into two quantum registers: one is the address register with $n=\lceil \log_{2}N\rceil$ qubits that can record the addresses for at least N nodes, and the other one is a register with q qubits used to store the embedding vectors. Overall, our circuit consists of three main parts: (i) n Hadamard gates used to prepare a uniform superposition in the address register, (ii) parametric quantum encoder and (iii) measurement layer. In the following section, we focus on parts (ii) and (iii).

### 2.1 Parametric quantum encoder

The parametric quantum encoder part is composed of m layers, where each layer comprises an encoding layer and mixing layer. In each encoding layer, there are n control-U operations with different parameters. The unitary operations can be varied with different control qubits but possess the same configuration in different layers. Then, under the control of a state $|p_{0}p_{1}\cdots p_{n}\rangle$, which marks the node $p={\bar{p_{0}p_{1}\cdots p_{n}}}_{\left(2\right)}$, the embedding register is affected by a unitary evolution $\prod_{j}{\left(U_{j}\left(\theta_{ij}\right)\right)}^{p_{j}}$.

If we only recurrently implement this operation m times in the initial state under our settings, this PQC ansatz performs well but does not account for the increasing depth. This is because the duplicated operation structures for the same control state limit the expressive power of the circuit, which should increase with the number of parameters. From an extreme perspective, we can assume that all $U_{j}$ are commutative to each other, and that the repeated layers will never behave better than one layer. Therefore, we must add layers, called mixing layers, between the encoding layers to alter their impact from the same control state.

In the mixing layer, we use quantum gates such as CNOT and X gates, which can maintain each encoding state with the same weight in the final state. Each mixing layer acts as a mapper that maps the control state |p〉 to another state, denoted as $|f_{i}\left(p\right)\rangle$. We define the control state after i maps as $F_{i}\left(p\right)$, which is equal to $f_{i}\circ f_{i-1}\circ \cdots \circ f_{1}\left(p\right)$ and $f_{0}\left(p\right)=p$. The encoded result for the initial control state |p〉 is given as

(6) $$|p\rangle |0\rangle \overset{U_{\text{QENC}}}{\to }|F_{m}\left(p\right)\rangle \prod_{i=1}^{m}\prod_{j=1}^{n}{\left(U_{j}\left(\theta_{ij}\right)\right)}^{{\left[F_{i-1}\left(p\right)\right]}_{j}}|0\rangle$$

The symbol ${\left[\cdot \right]}_{j}$ denotes the jth bit of the binary form of the number.

As the number of encoding layers and parameters increases, the representation space of the parameterized quantum encoder also increases; thus, it can capture more node relations. In other words, a circuit with N parameters is highly capable of capturing all information about the graph with N nodes, but such settings will lose the quantum advantage and are expensive to run on quantum hardwares. In our algorithm, we heuristically set the encoding layer number on the order of O(poly(n)), which allows the parameterized quantum encoder to capture only the major relations between nodes. In general, a group of embedding vectors that contain the main information of the graph is always sufficient for follow-up machine learning tasks. As n approximates to $\log_{2}N$, the complexity of our quantum encoder is O(poly(log(N),q)) by choosing $U_{j}$, which is composed of O(poly(q)) basic quantum gates.

### 2.2 Measurement layer

With a quantum encoder, we can produce a parameterized quantum embedding state |ψ(θ)〉. The following task is to optimize the parameters to obtain the maximum of L(θ). Take gradient-based optimization methods as an example; in each step of parameter updating, the gradient $\partial L(\theta )/\partial \theta_{i}$ is calculated according to $(L\left(\theta +\Delta \theta_{i}\right)-L\left(\theta -\Delta \theta_{i}\right))/\Delta \theta /2$, either directly or through parameter shift methods [29], [30]. Hereafter, we denote $\Delta \theta_{i}$ as a vector with all zero elements except the ith component, which is Δθ. In fact, no matter what optimization methods are used, we need to measure the expectation of M in Eq. 5 on the quantum state |ψ(θ)〉.

As usual, we can diagonalize the matrix as $M=U^{†}\Lambda U$. Then, we make the transformation U on the state |ψ(θ)〉 and obtain the expectation on the probability distribution from the measurement in the computation basis because $\langle M\rangle =\sum_{i}\Lambda_{ii}{\left|\langle i|U|\psi (\theta )\rangle \right|}^{2}$. However, the diagonalization process consumes a numerous classical computational resources. Moreover, the evolution of U may have enormous gate complexity and thus require significant quantum computational resources.

In our scheme, we firstly decompose M into a linear combination of Pauli product terms as $M=\sum_{i}\alpha_{i}P_{i}$ and measure the expectation of each Pauli product $\langle P_{i}\rangle$. The resources for the decomposition process and each $\langle P_{i}\rangle$ measurement are bearable. However, there are $O(4^{n})$ Pauli product terms in M in the worst case scenario, and such a direct method complicates the QNE algorithm and loses its advantage. Fortunately, some of the Pauli products’ expectation can be estimated under the same basis simultaneously by applying the same unitary transformation to |ψ(θ)〉. Therefore, we can group the Pauli product operators into several sets and significantly reduce the number of measurements required. Specifically, we must evaluate 〈A〉 and $\langle J_{2^{n}}\rangle$ to calculate L(θ), where $\langle \psi |\cdot ⊗I^{⊗q}|\psi \rangle$ is simply denoted as 〈·〉 hereafter. For operator $J_{2^{n}}={\left(I+X\right)}^{⊗n}$, by applying $H^{⊗n}$ on the quantum state |ψ(θ)〉 to get $|\psi^{\prime }\left(\theta \right)\rangle$, we can obtain $\langle \psi |J_{2^{n}}⊗I^{⊗q}|\psi \rangle$ by $\langle \psi^{\prime }|{\left(I+Z\right)}^{⊗n}⊗I^{⊗q}|\psi^{\prime }\rangle$, where ${\left(I+Z\right)}^{⊗n}$ is equal to $2^{n}{|0\rangle }_{n}{\langle 0|}_{n}$, which can be reduced to obtaining the probability of the measurement result that all qubits in the address register are zero. For a more general Hermitian matrix A, we can first decompose A into $\sum_{p,q}A^{pq}$ while $A^{pq}$ is a $2^{n}\times 2^{n}$ matrix with all elements being zero, but the element in the pth row and qth column is equal to one. We then prove that the expectation of the matrix $A^{pq}+A^{qp}$, which represents the edge between nodes p and q, can be obtained by performing a unitary transformation with O(n) basic gates. Therefore, the gate complexity for obtaining one count when measuring 〈A〉 is O(dn), where d is the number of edges in the graph; the details of the derivation are given in Supplementary Section 3. Thus, we can conclude that our QNE algorithm works well for sparse graphs.

For a dense graph, the measurement complexity can be reduced using two heuristic methods. One measures the expectation of operator $J_{2^{n}}-A$ first, which is sparser, and then computes 〈A〉 by $-\langle \left(J_{2^{n}}-A\right)\rangle +\langle J_{2^{n}}\rangle$. The other approach considers several edges simultaneously as a special structure instead of considering only one edge at a time. For instance, a subgraph, which is a chain linking several nodes in sequence, leads to a simple Pauli product summation and therefore reduces the measurement complexity, as shown in Supplementary Section 4.

Another factor to consider is the number of measurement repetitions to obtain the term 〈M〉. From the central limit theorem, the approximate number should be $O({\left(\Delta M\right)}^{2}/\epsilon^{2})$ where ΔM denotes the standard deviation of measurement results and ϵ is the accuracy. Because it is difficult to determine the variance in advance, the exact number of measurement before the experiments are completed is unknown in many quantum-classical hybrid algorithms. However, we only need to keep increasing the number of measurements until the final result converges to the desired level. It should be emphasized that our task is to provide a quantum embedding state rather than solve the ground state problem. Therefore, even when the objective function does not reach the global maximum, the corresponding parameterized quantum state can still reflect the main properties of the graph well. As Fig. 2 shows, the measurement layer consists of some basic single- or two-qubit quantum gates. When we consider the high-order correlation similarity function rather than an adjacency matrix, more complicated quantum gates in the measurement layer should be utilized; more details are discussed in the next subsection.

### 2.3 High-order relation similarity function

In the previous subsection, we set the similarity function as an adjacency matrix A. However, the matrix A captures only the relations between two nodes linked by an edge. In other words, indirectly connected relations between two nodes through other intermediate nodes are not considered. To reveal additional relation information between two nodes in the embedding state, a high-order similarity function should be considered.

The high-order relation defined here refers to the connection of nodes through other nodes, which differs from the first-order relation in which two nodes connect directly. Taking the square of the adjacency matrix A as an example, the element $A^{2}\left[i,j\right]$, which is in the ith row and jth column of $A^{2}$, is equal to $\sum_{k}A_{ik}A_{kj}$, which indicates the accumulation of all paths from node-i to node-j passing through only one node. Then, $\langle A^{2}\rangle$ can be used as a measure of the second-order neighborhood information. Similarly, we can also construct $A^{3},A^{4}\cdots$ for higher-order relations. If we combine these items to form an overall measure, a decay factor β must be added to readjust their contributions.

For a simpler implementation on a quantum computer, we provide two types of measurement operators that can be used as replacements for A in Eq. 5. The first is

(7) $${\left(I-\beta A\right)}^{-1}=\sum_{i=0}^{\infty }\beta^{i}A^{i}$$

which holds when $|\beta \cdot \lambda_{\max}|<1$. (I−βA) is a Hermitian matrix and $\lambda_{\max}$ is the eigenvalue of A with the largest absolute value. We could first use the quantum algorithm for the linear system [4] to produce the quantum state $|\psi^{\prime }\rangle =\frac{1}{\sqrt{C}}{\left(I-\beta A\right)}^{-1}|\psi \rangle$ and its normalization factor C. After producing |ψ〉 and $|\psi^{\prime }\rangle$, we can use the Hadamard-test algorithm [31] to get $\langle \psi |\psi^{\prime }\rangle$ with the aid of an ancilla qubit. Then, $\langle {\left(I-\beta A\right)}^{-1}\rangle$ can be obtained as $\sqrt{C}\langle \psi |\psi^{\prime }\rangle$. The other high-order similarity measure is

(8) $$e^{\beta A}=\sum_{n=0}^{\infty }\beta^{n}A^{n}/n!$$

$\langle e^{\beta A}\rangle$ can be obtained with the quantum imaginary time evolution algorithm [32]. The implementation details of these two methods are provided in Supplementary Section 2. In this manner, we extend our algorithm to higher-order cases without compromising its feasibility.

## 3 Experiment

We demonstrated our QNE algorithm on a real NMR quantum processor. The liquid NMR system uses spins as qubit registers, whose Hamiltonian is $H=\sum_{i}\pi \nu_{i}\sigma_{z}^{i}+\sum_{i<j}\frac{\pi }{2}J_{ij}\sigma_{z}^{i}\sigma_{z}^{j}$. The experimental sample consists of $C^{13}$-labeled trans-crotonic acid dissolved in d6-acetone. The trans-crotonic acid molecule includes four carbons, two hydrogens, and one methyl group, and can be regarded as a 7-qubit quantum information processor. By decoupling the hydrogen nuclei from the carbons, the remaining carbon spins become a 4-qubit homonuclear system. The hydrogen nuclei were decoupled using standard heteronuclear decoupling techniques. Our experiment was performed on a Bruker Avance III 400 MHz spectrometer at a temperature of 300 K. The structure and Hamiltonian parameters of the molecule are presented in Supplementary Section 5. In this four-qubit system, we prepared I⊗|000〉〈000| as the initial state, with its fidelity of more than 99.5%. In the subsystem of $C_{2},C_{3},C_{4}$, we get a pseudo pure state |000〉, which is the average of an ensemble with a huge number of molecules.

**Fig. 3:**
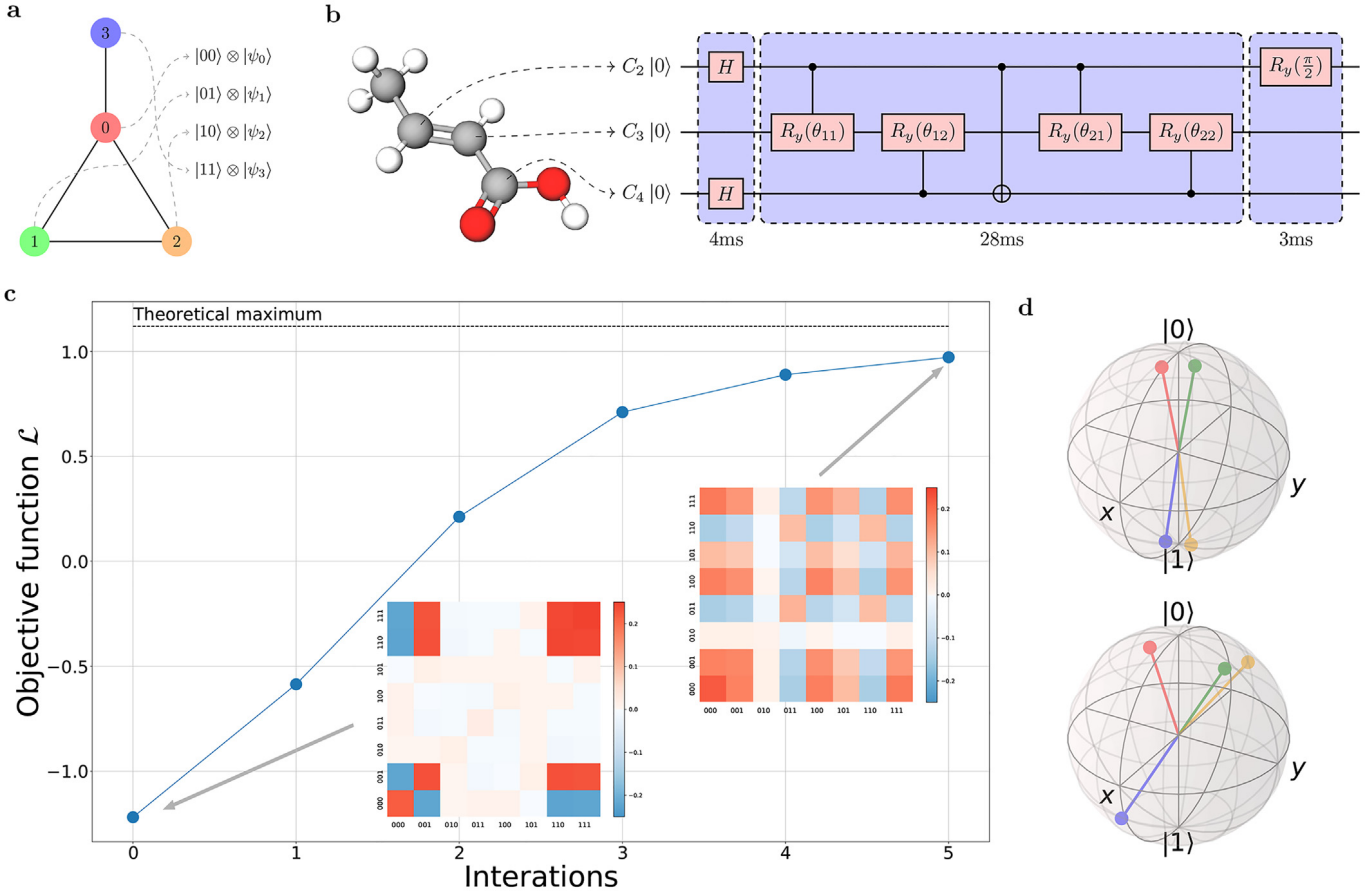
**Experimental settings and results.** (a) Node graph for the experiment. (b) The molecular structure of the NMR processor, the experimetal quantum circuit and their correspondence. The quantum circuit is divided into three parts, which are packaged into individual pulse sequences with time lengths of 4 ms, 28 ms and 3 ms. (c) Objective function L in each iteration. The subfigures shown in the inset are the real parts of the experimental density matrix at the start and end of the iteration. (d) Embedding states for four nodes displayed in a Bloch sphere. The top one is in iteration-0, while the bottom one is in iteration-5.

The node graph to be embedded in the quantum state is shown in Fig. 3a. The quantum embedding state involves the last three qubits of the 4-qubit quantum information processor as a working system, where two qubits ($C_{2}$, $C_{4}$) are used as the address register, which represents four nodes, and the remaining qubit ($C_{3}$) encodes the embedding vectors, as shown in Fig. 3b. The quantum circuit comprises three parts: the first consists of two Hadamard gates to produce a uniform superposition in the address register, the second is a PQC with two encoding layers and one mixing layer, and the last is the measurement layer. In this experiment, we chose $R_{y}\left(\theta \right)$ as the $U_{j}\left(\theta \right)$ in Eq. 6 for quantum hardware efficiency. To achieve a higher fidelity, we packaged each part into an 8×8 unitary operator U and used the technique of gradient ascent pulse engineering [33] to calculate the control pulse sequence of I⊗U in this 4-qubit quantum system. This means that there is no final effect on $C_{1}$ nuclear spin, but we must account for it in the calculation of the pulse because $C_{1}$ has a Larmor frequency similar to that of other carbon nuclear spins and couples with them. The evolution times for each part were 4 ms, 28 ms, and 3 ms, respectively, as shown in Fig. 3b. The second part had a much longer duration because the coupling strength between $C_{2}$ and $C_{4}$ is weak. All calculated pulses had a fidelity of more than 99.5% and were robust to the inhomogeneity of the radio-frequency pulses in the calculation.

For the measurement of the Pauli product series, the items in the objective function $L(\theta )=\langle A-\gamma (J_{4}-A)\rangle$ are:

(9) $$A=\left[\begin{array}{cccc} 0 & 1 & 1 & 1\\ 1 & 0 & 1 & 0\\ 1 & 1 & 0 & 0\\ 1 & 0 & 0 & 0 \end{array}\right],J_{4}=\left[\begin{array}{cccc} 1 & 1 & 1 & 1\\ 1 & 1 & 1 & 1\\ 1 & 1 & 1 & 1\\ 1 & 1 & 1 & 1 \end{array}\right],$$

where A can be decomposed as 0.5*(I⊗X+Z⊗X+X⊗I+X⊗Z)+X⊗X, and $J_{4}$ can be decomposed as ${\left(I+X\right)}^{⊗2}$. We can construct our measurement layers by measuring the expectation of the Pauli operators {IIIX,IZIX,IXII,IXIZ,IXIX} in our 4-qubit quantum system. In the NMR quantum system, we perform measurements by collecting the radiation signal in the XY-plane of a system of each spin and performing a Fourier transformation on the time-varying signal. From the spectrum of the ith carbon nuclear spin, we can obtain $\langle X_{i}⊗|d\rangle \langle d|\rangle$, where |d〉 belongs to the computation basis of the other three qubits. Thus, only two measurement operators are required in our measurement layers: One is the identity operator, since we can obtain 〈IIIX〉 and 〈IZIX〉 from the spectrum of $C_{4}$ and 〈IXII〉, 〈IXIZ〉 from the spectrum of $C_{2}$ directly without any rotation operation. The other one is a rotation $R_{y}\left(\frac{\pi }{2}\right)$ on $C_{2}$, through which we can obtain 〈IXIX〉 from the spectrum of $C_{4}$.

## 4 Results

We set the hyperparameter γ=0.5 for the objective function, Δθ=π/10 for calculating gradient, and the learning rate α=2 for updating θ. Before the experiment started, for the convenience of performing the first iteration of experiments, all θ components were initialized at π as our first guess. In each iteration, we calculated the gradient by running the quantum circuit and updating θ for the next iteration. The experimental data of this process are presented in Supplementary Section 5. At the end of the iteration, the objective function converges, as shown in Fig. 3c. The optimized parameters and maximal values of the objective function were obtained. To clearly demonstrate the node embedding algorithm, we performed quantum state tomography at the start and end of the iterations, where the ideal final density matrix ρ of the 4-qubit system is given by Eq. 10:

(10) $$\rho =\sum_{i,j,m,n=0}^{1}I⊗|i\rangle \langle j|⊗|x_{im}\rangle \langle x_{jn}|⊗|m\rangle \langle n|$$

By tracing out $C_{1}$’s part, we can obtain the 8×8 density matrix for the subsystem of $C_{2},C_{3},C_{4}$ in the experiment. Obviously, ρ in the iteration process only have real parts in theory, and the real parts of the subsystem density matrix in the experiment at the start and end of the iterations are presented in the inset of Fig. 3c.

By defining $U_{im}=I⊗|i\rangle \langle i|⊗I⊗|m\rangle \langle m|$, we can extract the density matrix $\rho_{2i+m}$ of the embedding register ($C_{3}$) corresponding to the (2i+m)th node by partial trace ${\text{tr}}_{1,2,4}\left(U_{im}\rho U_{im}\right)$. Then, we can present all density matrices of $\rho_{2i+m}$, namely, $\rho_{0},\rho_{1},\rho_{2},\rho_{3}$ in the Bloch sphere in Fig. 3d. In the top panel, the four nodes converge into two piles in the initial stage (iteration-0). In the bottom panel (iteration-5), nodes 1 and 2 separate from nodes 0 and 3, respectively, and become closer to each other. Moreover, both nodes 1 and 2 are closer to node 0 than to node 3. Therefore, after the iterations of our QNE algorithm, the geometry of the density matrix in the Bloch sphere clearly reveals the topological relations between the nodes in the graph in Fig. 3a.

**Fig. 4:**
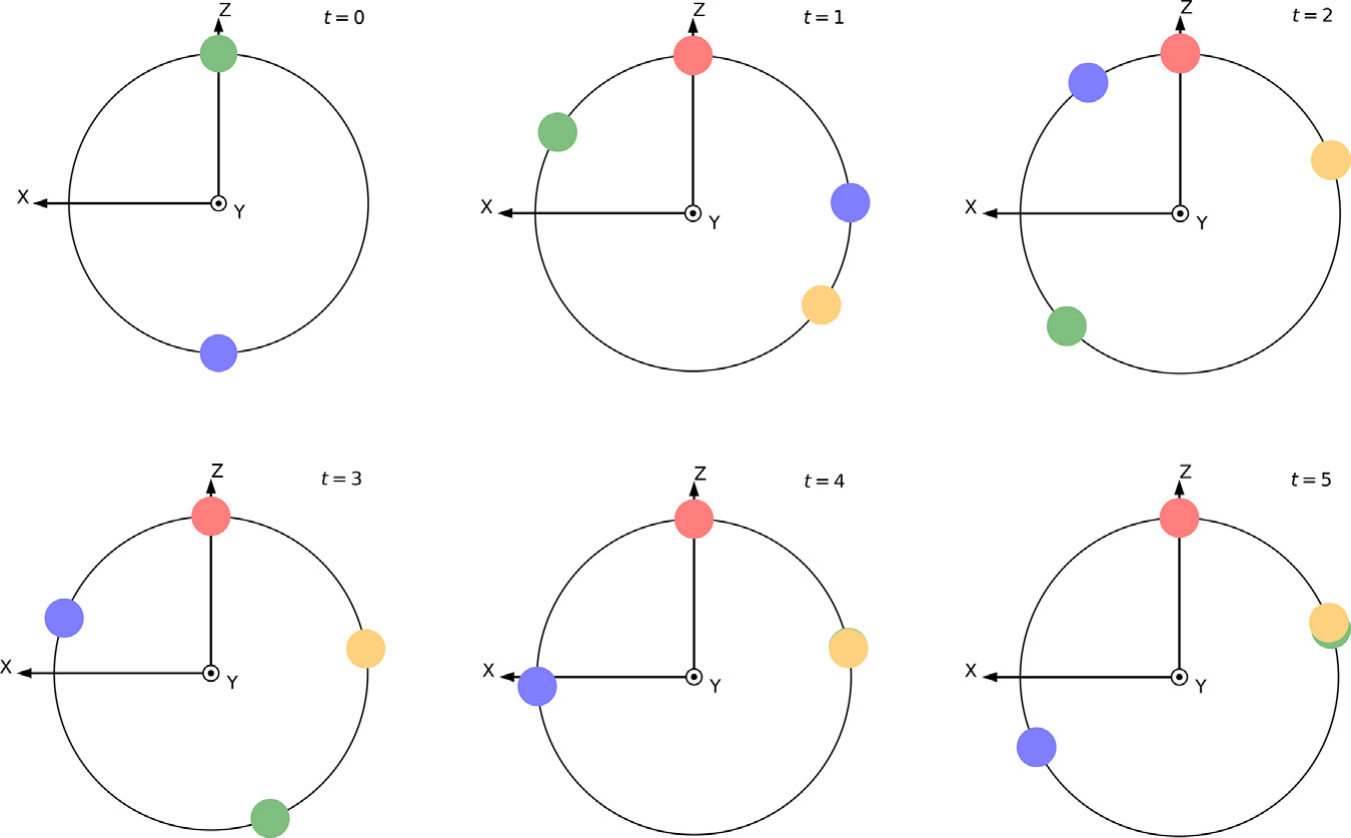
**Change of node states in the training process**. The theoretical embedding states are illustrated in the XZ-plane of the Bloch sphere during iterations, which are calculated by substituting the parameter vector θ obtained from the experimental result into our parametric quantum circuit. The red, green, yellow, and blue circles denote nodes 0 to 3, respectively. (For interpretation of the references to colour in this figure legend, the reader is referred to the web version of this article.)

To give a clearer perspective, we calculated the theoretical embedding state in each iteration by substituting the parameter vector θ obtained from the experimental result into our parametric quantum circuit. In each iteration, we extracted the embedding vector for each node and illustrate them in the XZ-plane of the Bloch sphere, as shown in Fig. 4. We can see that these four nodes change from two piles at iteration t=0 to three piles at iteration t=5, where nodes 2 and 3 share almost the same coordinates and are closer to node 0 than to node 3. This indicates that our experiment accurately captured this iterative process.

## 5 Conclusion

In summary, we developed and experimentally implemented a quantum algorithm for node embedding, that can produce a quantum embedding state with classical information about the graph as input. The resulting state of our algorithm can be used as an input for other QML algorithms, such as quantum clustering algorithms. Owing to the use of the PQC with O(poly(log(N))) gates in producing the state, one can repeatedly prepare the quantum embedding state for N nodes with low complexity just as QRAM does. Furthermore, we proposed a general method for reducing measurement complexity and heuristic methods for further reduction. In addition, we discussed capturing high-order relations, which makes this algorithm more applicable. Our algorithm can also play a significant role in other quantum computing systems, such as superconducting circuits, ion traps, and neutral atoms.

## CRediT authorship contribution statement

**Zeng-rong Zhou:** Conceptualization, Data curation, Formal analysis,Methodology, Software, Visualization, Writing – original draft. **Hang Li:** Formal analysis, Funding acquisition, Investigation, Project administration, Software, Supervision, Visualization, Writing – original draft. **Gui-Lu Long:** Funding acquisition, Investigation, Resources, Supervision, Validation, Writing – review & editing.

## Declaration of competing interest

The authors declare that they have no conflicts of interest in this work.

## Appendix A Supplementary materials

Supplementary Data S1 Supplementary Raw Research Data. This is open data under the CC BY license http://creativecommons.org/licenses/by/4.0/ Supplementary Data S1
