# Supplementary material for: Variational quantum algorithm for node embedding
Source: Fundam Res. 2023 Oct 14;4(4):845–50. doi: 10.1016/j.fmre.2023.10.001 (PMC11330110; doi:10.1016/j.fmre.2023.10.001)
Supplement: Supplementary Data S1 — Supplementary Raw Research Data. This is open data under the CC BY license http://creativecommons.org/licenses/by/4.0/ [file mmc1.pdf]

Supplementary Material of 'Variational quantum algorithm for node embedding'

Zeng-rong Zhou, Hang Li, and Gui-Lu Long

1. QUANTUM NODE EMBEDDING ALGORITHM

The main framework of our algorithm can be described as follows. Firstly, we set a quantum encoder circuit that can produce the state $|\psi(\boldsymbol{\theta})\rangle$. The $\boldsymbol{\theta}$ vector is initialized randomly and updated with each round. Then we choose one of three different algorithms for estimating the objective function $\mathcal{L}(\boldsymbol{\theta})$ which is the measurement result as $\langle M\rangle_{|\psi(\boldsymbol{\theta})\rangle}$. The main difference for those subroutines is the different measurement layers. We can update the parameters by repeatedly calling the process above. The natural language description of our algorithm's vanilla version, which only captures the first-order neighborhood information, is stated as Algorithm 1.

Algorithm 1: Vanilla Quantum Node Embedding Algorithm

Input: The $N \times N$ adjacency matrix A. Hyperparameter γ, α, $\Delta\theta$, q[a].
Output: $\frac{1}{\sqrt{2^n}} \sum_i |i\rangle |\mathbf{x}_i(\boldsymbol{\theta})\rangle$.

Extend A to a $2^n \times 2^n$ matrix by adding zero where $n = \lceil \log_2 N \rceil$.
Decompose $(A - \gamma(J_{2^n} - A))$ as $\sum_i S^{(i)}$ while every $S^{(i)} = \sum_j \alpha_j^{(i)} \mathcal{P}_j^{(i)}$[b].
Construct the measurement layers for every $S^{(i)}$.
repeat
  for each θ_i in $\boldsymbol{\theta}$ **do**
    Estimate $\mathcal{L}(\boldsymbol{\theta} + \Delta\boldsymbol{\theta_i})$ and $\mathcal{L}(\boldsymbol{\theta} - \Delta\boldsymbol{\theta_i})$ by `getL-1()`
    Calculate $\partial\mathcal{L}(\boldsymbol{\theta})/\partial\theta_i = (\mathcal{L}(\boldsymbol{\theta} + \Delta\boldsymbol{\theta_i}) - \mathcal{L}(\boldsymbol{\theta} - \Delta\boldsymbol{\theta_i}))/\Delta\theta/2$
  end
  Update parameter by $\boldsymbol{\theta} \leftarrow \boldsymbol{\theta} + \alpha \cdot \partial\mathcal{L}(\boldsymbol{\theta})/\partial\theta$.
  Estimate $\mathcal{L}(\boldsymbol{\theta})$ by `getL-1()`
until $\mathcal{L}(\boldsymbol{\theta})$ converges with changing $\boldsymbol{\theta}$;

Subroutine `getL-1()`
  Input: Parameter $\boldsymbol{\theta}$, Set $\{S^{(i)}\}$.
  Output: $\mathcal{L}(\boldsymbol{\theta})$
  repeat
    for every $S^{(i)}$ in set **do**
      1. Initialize the quantum state as $|0\rangle_n |0\rangle_q$.
      2. Apply quantum encoder circuit to the initial state to get $\frac{1}{\sqrt{2^n}} \sum_i |i\rangle |\mathbf{x}_i(\boldsymbol{\theta})\rangle$.
      3. Apply the measurement layer to address register and measure this register in the computation basis.
      4. Repeat step 1-3 for several times.
      5. Compute each $\langle \mathcal{P}_j^{(i)} \rangle$ based on the measurement result.
    end
    $\mathcal{L}(\boldsymbol{\theta})$ = weighted average of $\langle \mathcal{P}_j^{(i)} \rangle$.
  until $\mathcal{L}(\boldsymbol{\theta})$ converges with the growing measurement times;

[a] The q is the embedding register's qubit number.
[b] All $\mathcal{P}_j^{(i)}$ with same i can be get from the measurement results under the same basis.

2. QUANTUM NODE EMBEDDING FOR HIGH-ORDER RELATION

From Eq. (5) in the main manuscript, we renormalize M as $(A - \gamma' J_{2^n}) \otimes I$ by extracting a constant. In this expression, we only use the fisrt-order neighborhood information. For capturing high order relations in a graph, we can replace A with a high-order similarity measure. We have two options in our algorithm while the first one is $\sum_{i=0}^{\infty} \beta^i A^i$ which contains the relation at all levels and renormalizes them by a decay factor β. Now as Theorem 1 shows, we could convert $\sum_{i=0}^{\infty} \beta^i A^i$ to $(I - \beta A)^{-1}$ under the condition of $|\lambda_{max} \cdot \beta| < 1$ where λ_{max} is the eigenvalue of A with the largest absolute value.

Algorithm 2: Quantum Node Embedding with Matrix-inverse Method

Input: The adjacency matrix A. Hyperparameter γ, α, $\Delta\theta$, β, q.
Output: $\sum_i |i\rangle |\mathbf{x}_i\rangle$

```
Set the qubit number of address register as n = ⌈log_2 N⌉. The second register is embedding with qubit num m.
Constuct the e^{-i(I-βA)t}
repeat
    for each θ_i in θ do
        Estimate L(θ + Δθ_i) and L(θ − Δθ_i) by getL-2()
        Calculate ∂L(θ)/∂θ_i = (L(θ + Δθ_i) − L(θ − Δθ_i))/Δθ/2
    end
    Update parameter by θ ← θ + α · ∂L(θ)/∂θ.
    Estimate L(θ) by getL-2()
until L(θ) converges with changing θ;

Subroutine getL-2()
    Input: Parameter θ.
    Output: L(θ)
    1. Initialize the quantum state as |0⟩_n |0⟩_q.
    2. Apply quantum encoder circuit to the initial state to get 1/√(2^n) Σ_i |i⟩ |x_i(θ)⟩.
    3. Apply matrix-inverse evolution to the address register of the final state to get |ψ'⟩ = 1/√C (I − βA)^{-1} |ψ⟩ and C.
    4. Run the Hadamard test algorithm for estimating ⟨ψ'|ψ⟩.
    5. Repeat step 1-4 for several times to get L(θ) = √C · ⟨ψ'|ψ⟩
```

Theorem 1 *Let A be a Hermitian matrix and its absolutely largest eigenvalue is λ_{max}. Then*

$$(I-\beta A)^{-1} = \sum_{i=0}^{\infty} \beta^i A^i \tag{S1}$$

if $|\lambda_{max} \cdot \beta| < 1$ and $(I-\beta A)$ is non-singular.

Proof 1 *Let $S_n = \sum_{i=0}^{n} \beta^i A^i$, then we have*

$$\beta A S_n = \sum_{i=1}^{n+1} \beta^i A^i \tag{S2}$$

so,

$$\begin{aligned}(I-\beta A)S_n &= \sum_{i=0}^{n} \beta^i A^i - \sum_{i=1}^{n+1} \beta^i A^i \\ &= I - \beta^{n+1} A^{n+1}\end{aligned} \tag{S3}$$

since $(I-\beta A)$ is non-singular, we have,

$$\begin{aligned}\sum_{i=0}^{\infty} \beta^i A^i &= \lim_{n\to\infty} S_n \\ &= \lim_{n\to\infty} (I-\beta A)^{-1}(I-\beta^{n+1}A^{n+1}) \\ &= (I-\beta A)^{-1}\end{aligned} \tag{S4}$$

The derivation of the last step is based on $\lim_{n\to\infty} \beta^n A^n = \lim_{n\to\infty} \sum_i (\lambda_i \beta)^n \xi_i \xi_i^T = 0$ where $|\lambda_{max} \cdot \beta| < 1$.

Then, if we can estimate the expectation value $\langle (I-\beta A)^{-1} \rangle$, we could get the objective function $\mathcal{L}(\theta)$. The main framework of this process can be stated as follows in which we use matrix-inverse method from A.W.Harrow et al. [1]. It's a brief review of the method and its adaptation to our algorithm. If you want to know more about the detailed implemtation and demonstration, please refer to the original article. Firstly, we construct the unitary operator $e^{i(I-\beta A)\Delta\tau}$ which will be efficiently implemented in quantum computer while A is a sparse matrix. Denote

λ_k and $\{|e_k\rangle\}$ as the eigenvalues and eigenvectors of $I - \beta A$. Then from a clearer perspective, we can expand each position vector $|i\rangle$ in $|\psi(\theta)\rangle = \frac{1}{\sqrt{2^n}} \sum_i |i\rangle |\mathbf{x}_i(\theta)\rangle$ under the basis vector set $\{|e_k\rangle\}$. So, the state $|\psi(\theta)\rangle$ can be viewed as linear combination of those mutually orthogonal state $|e_k\rangle |\mathbf{x}_i(\theta)\rangle$. For every $|e_k\rangle |\mathbf{x}_i(\theta)\rangle$, applying the operator $(I - \beta A)^{-1} \otimes I^{\otimes q}$ on it will result in a factor $\frac{1}{\lambda_k}$ before state. We can implement this effect equivalently through the following steps. By applying quantum phase estimation algorithm [2] with the assistant of $e^{i(I-\beta A)\Delta\tau}$, we can generate the approximate eigenvalue λ_k in ancilla register and the state comes to $|\lambda_k\rangle |e_k\rangle |\mathbf{x}_i(\theta)\rangle$. With a control rotation as $|\lambda\rangle \langle\lambda| \otimes R_y(\arccos(\lambda^{-1}))$ implementing on an ancilla qubit, we can get state$|\lambda_k\rangle |e_k\rangle |\mathbf{x}_i(\theta)\rangle \left(\frac{1}{\lambda_k} |0\rangle + \sqrt{1 - \frac{1}{\lambda_k^2}} |1\rangle\right)$. Measuring the ancilla qubit, post-selecting the state with result $|0\rangle$ and uncomputing the former operations, we can get $\frac{1}{\lambda_k} |e_k\rangle |\mathbf{x}_i(\theta)\rangle$. All mutually orthogonal quantum states $|e_k\rangle |\mathbf{x}_i(\theta)\rangle$ are applied the same set of operations and therefore go through a $(I - \beta A)^{-1}$ evolution. In summary, with an input state $|\psi\rangle$, we can get the state $|\psi'\rangle$ as,

$$|\psi'\rangle = \frac{1}{\sqrt{C}} (I - \beta A)^{-1} \otimes I^{\otimes q} |\psi\rangle \tag{S5}$$

and the value of normalization factor C. Now, with the ability of producing state $|\psi\rangle$ and $|\psi'\rangle$, we could implement Hadamard-test algorithm [3] to evaluate the value $\langle\psi|\psi'\rangle$ which must be real in our algorithms.

$$\langle\psi| (I - \beta A)^{-1} \otimes I^{\otimes q} |\psi\rangle = \sqrt{C} \langle\psi|\psi'\rangle \tag{S6}$$

Finally, we get the $\langle (I - \beta A)^{-1} \rangle$ with the matrix-inverse evolution method at cost of $O(n\kappa^2 d^2/\epsilon)$ gates. Here, κ is the condition number of A, d is the sparsity of A and ϵ is the operation error. The natural language description of the matrix-inverse version of our algorithm is stated as Algorithm. 2.

Algorithm 3: Quantum Node Embedding with QITE Method

Input: The adjacency matrix A. Hyperparameter γ, α, $\Delta\theta$, β.
Output: $\frac{1}{\sqrt{2^n}} \sum_i |i\rangle |\mathbf{x}_i\rangle$

Set the qubit number for address register as $n = \lceil \log_2 N \rceil$. The second register is embedding with qubit num m.
repeat
  for each θ_i in θ **do**
    Estimate $\mathcal{L}(\theta + \Delta\theta_i)$ and $\mathcal{L}(\theta - \Delta\theta_i)$ by `getL-3()`
    Calculate $\partial\mathcal{L}(\theta)/\partial\theta_i = (\mathcal{L}(\theta + \Delta\theta_i) - \mathcal{L}(\theta - \Delta\theta_i))/\Delta\theta/2$
  end
  Update parameter by $\theta \leftarrow \theta + \alpha \cdot \partial\mathcal{L}(\theta)/\partial\theta$.
  Estimate $\mathcal{L}(\theta)$ by `getL-3()`
until $\mathcal{L}(\theta)$ converges with changing θ;

Subroutine `getL-3()`
  Input: Parameter θ.
  Output: $\mathcal{L}(\theta)$
  1. Initialize the quantum state as $|0\rangle_n |0\rangle_m$.
  2. Apply quantum encoder circuit to the initial state to get $\frac{1}{\sqrt{2^n}} \sum_i |i\rangle |\mathbf{x}_i(\theta)\rangle$. Set as $|\psi_0\rangle$.
  for $i = 0$ to $n - 1$ **do**
    if $i == 0$ **then**
      $|\psi_i\rangle = |\psi_0\rangle$
    else
      Building the $|\psi_i\rangle$ from gates construction recorded before.
    end
    Implemt QITE algorithm to get $|\psi_{i+1}\rangle = \frac{1}{\sqrt{C_i}} e^{\frac{1}{2}A\Delta t} |\psi_i\rangle$ from $|\psi_i\rangle$.
    Record the gates construction on building $e^{i\mathcal{P}\Delta\tau}$ and the normalization factor C_{i+1}.
  end
  $\mathcal{L}(\theta) = C_1 C_2 \cdots C_n$

Another similarity measure for high-order relation is $e^{\beta A}$ whose Taylor expansion is $\sum_{n=0}^{\infty} \beta^n A^n/n!$ which contains all order relations and renormalizes them by a $\beta^n/n!$ factor. Then our goal is to measure the expetation of this operator on the embedding state $|\psi\rangle$, which is shown in Eq. (S7).

$$C = \langle\psi| e^{\beta A} \otimes I^{\otimes q} |\psi\rangle \tag{S7}$$

Fortunately, this expectation can be evaluated by quantum imaginary time evolution algorithm (QITE) [4]. Next we will introduce its main framework. Our goal is to get the normailized fact C in Eq. (S8) which shows a state that can be viewed by n small steps imaginary time evolution. Δt is equal to β/n in the equation while n is large enough for eliminating errors in each step.

$$|\psi_f\rangle = \frac{1}{\sqrt{C}} e^{\frac{1}{2}\beta A} \otimes I^{\otimes q} |\psi\rangle = \frac{1}{\sqrt{C_n}} e^{\frac{1}{2}A\Delta t} \frac{1}{\sqrt{C_{n-1}}} e^{\frac{1}{2}A\Delta t} \cdots \frac{1}{\sqrt{C_1}} e^{\frac{1}{2}A\Delta t} |\psi\rangle . \tag{S8}$$

According to the QITE algorithm and as Eq. (S9) shows, every step from $|\psi_i\rangle$ to $|\psi_{i+1}\rangle$ can be replaced by a unitray transformation with the same effect,

$$|\psi_{i+1}\rangle = \frac{1}{\sqrt{C_i}} e^{\frac{1}{2}A\Delta t} |\psi_i\rangle = e^{i\mathcal{P}\Delta\tau} |\psi_i\rangle . \tag{S9}$$

In which $\mathcal{P}$ is the linear combination of Pauli product as $\alpha_i \mathcal{P}_i$ while the coefficients can be get from solving linear equations. Executing the n processes in sequence, we will get the final C as which equal to the value in Eq. (S7),

$$C = C_n C_{n-1} \cdots C_1. \tag{S10}$$

The gate complexity in every small step comes from the basic quantum gates to construct the evolution $e^{i\mathcal{P}\Delta\tau}$, which is relevant to the properties of matrix A. Just like the QITE algorithm, our algorithm will benefit quantum advantages while A can be decomposed as local terms and has a small relation length.

3. MEASUREMENT OPERATOR DECOMPOSITION

In order to practically measure the expectation of a Hermitian operator in a quantum state, we should decompose the operator into Pauli product series as $M = \sum_i \alpha_i \mathcal{P}_i$. And then we can measure every $\langle \mathcal{P}_i \rangle$ by transforming them to $\{I, Z\}^{\otimes n}$ basis as $\langle U^\dagger (I \otimes Z \cdots \otimes Z) U \rangle$ and sum them all with weights to get $\langle M \rangle = \sum_i \alpha_i \langle \mathcal{P}_i \rangle$. Now, we introduce a general approach to the decomposition problem, which serves as a subroutine for our improved algorithm. First, we can define four matrices as,

$$S_{00} = \begin{bmatrix} 1 & 0 \\ 0 & 0 \end{bmatrix} = \frac{1}{2}(I + Z) \tag{S11a}$$

$$S_{01} = \begin{bmatrix} 0 & 1 \\ 0 & 0 \end{bmatrix} = \frac{1}{2}(X + iY) \tag{S11b}$$

$$S_{10} = \begin{bmatrix} 0 & 0 \\ 1 & 0 \end{bmatrix} = \frac{1}{2}(X - iY) \tag{S11c}$$

$$S_{11} = \begin{bmatrix} 0 & 0 \\ 0 & 1 \end{bmatrix} = \frac{1}{2}(I - Z) \tag{S11d}$$

As Fig. 1 shows, for every matrix with shape $2^n \times 2^n$, we can decompose it as the summation of tensor product of S and a $2^{n-1} \times 2^{n-1}$ matrix. We can do this process iteratively and finally get the Pauli product series of the measurement

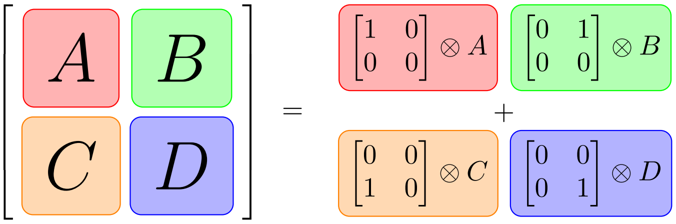

Supplementary Figure 1: **Matrix operator decompostion.** The $2^n \times 2^n$ matrix is decomposed as the summation of tensor product of S and a $2^{n-1} \times 2^{n-1}$ matrix.

operator. Next, we turn to practical cases where we must decompose A and J_{2^n} for efficient measurement. For the operator J_{2^n}, we can easily decomposite it as $(I + X)^{\otimes n}$. Then we can choose an operator from the Clifford group

as Eq. (S12) to change those measurement operators to another summation of the Pauli product series, which can be estimated with measurement results in $\{|0\rangle, |1\rangle\}^{\otimes n}$ basis.

$$(I+X)^{\otimes n} = H^{\otimes n}(I+Z)^{\otimes n}H^{\otimes n} \tag{S12}$$

So, $\langle\psi| ((I+X)^{\otimes n}\otimes I^{\otimes q}) |\psi\rangle$ is equal to $\langle\psi'| (I+Z)^{\otimes n}\otimes I^{\otimes q} |\psi'\rangle$ while $|\psi'\rangle = H^{\otimes n}\otimes I^{\otimes q}|\psi\rangle$. By applying $H^{\otimes n}\otimes I^{\otimes q}$ to $|\psi\rangle$ produced by the quantum encoder circuit, doing measurement in $\{|0\rangle, |1\rangle\}^{\otimes n}$ basis and repeating these processes, we can get the probability distribution in Z basis. So here we only need to get the probability of state vector $|00\cdots0\rangle$ in the address register because $(I+Z)^{\otimes n} = 2^n |00\cdots0\rangle\langle 00\cdots0|$.

The adjacency matrix A, which is generally a Hermitian matrix, can be expanded as $A = \sum_{p,q} A^{pq}$. A^{pq} here denotes a matrix with all elements equal to zero except the element in pth row and qth column, which is equal to one. The labels p and q can be written in binary string format as $p = \overline{p_0p_1\cdots p_n}_{(2)}$ and $q = \overline{q_0q_1\cdots q_n}_{(2)}$. Then the matrix A^{pq} can be expanded as Eq. (S13) with the aid of the method introduced at the beginning of this section,

$$A^{pq} = S_{p_0q_0}\otimes S_{p_1q_1}\otimes S_{p_2q_2}\cdots\otimes S_{p_nq_n}. \tag{S13}$$

While $p'q'$ pair equals to 00 or 11, the item $S_{p'q'}$ is a linear combination of I and Z. So we need to convert $S_{p'q'}$ to those same items when the $p'q'$ pair is equal to 01 or 10 by a unitary transformation. Here, one-qubit rotation does not work anymore. In Eq. (S14), we show that $S_{01/10}$ matrix in qubit i and qubit j can be transformed by applying a CNOT gate on the qubit pair.

$$\begin{aligned}
& \mathrm{CNOT}(i,j)(S_{01}^{(i)}\otimes S_{01/10}^{(j)})\mathrm{CNOT}(i,j) \\
= \;& \frac{1}{4}(|0\rangle\langle 0|\otimes I + |1\rangle\langle 1|\otimes X)((X+iY)\otimes(X\pm iY))(|0\rangle\langle 0|\otimes I + |1\rangle\langle 1|\otimes X) \\
= \;& \frac{1}{4}((X+iY)\otimes(X\pm iY))(|0\rangle\langle 0|\otimes I + |1\rangle\langle 1|\otimes X) \\
= \;& \frac{1}{4}(X+iY)\otimes(I\pm iYX) \\
= \;& \frac{1}{4}(X+iY)\otimes(I\pm Z) \\
= \;& S_{01}^{(i)}\otimes S_{00/11}^{(j)}
\end{aligned} \tag{S14}$$

In which we denote $\mathrm{CNOT}(i,j)$ as a CNOT gate with the ith qubit as the control qubit and the jth qubit as the controlled qubit. In the same way, we can get Eq. (S15),

$$\mathrm{CNOT}(i,j)(S_{10}^{(i)}\otimes S_{01/10}^{(j)})\mathrm{CNOT}(i,j) = S_{10}^{(i)}\otimes S_{11/00}^{(j)}. \tag{S15}$$

With those manipulation, the number of $S_{01/10}^{(j)}$ in measurement operator decreases by one. For a matrix A^{pq} with m items of $S_{01/10}$, we can use $m-1$ CNOT gates to reduce the number of items $S_{01/10}$ to one. For a matrix A^{qp}, we can apply the same transformations as A^{pq}. The transformation result of A^{pq} and A^{qp} only differ in one item that one is S_{01} and the other is S_{10}. As $S_{01}+S_{10}=X$, $A^{pq}+A^{qp}$, which represents an edge in the graph, can therefore be expressed as tensor product of $S_{00/11}$ except one item to be X. Then a single qubit gate such as $R_y(\frac{\pi}{2})$ on that qubit will make the whole operator become a summation of tensor products with I and Z whose expectations can be obtained from the measurement counts in the computation basis. For a graph with d edges, the measurement complexity for getting one count in measuring $\langle A\rangle$ is $O(dn)$. However, it is not enough in some situations. In the next part, there are some heuristic approaches for sophisticated cases.

4. HEURISTIC APPROACH

To accommodate more cases, we introduce two heuristic methods here to reduce the measurement complexity to a lower level. For a dense graph, we can replace $\langle A\rangle$ by $-\langle(J_{2^n}-A)\rangle + \langle J_{2^n}\rangle$. Here $J_{2^n}-A$ is a matrix sparser than A where J_{2^n} is equal to $(I+X)^{\otimes n}$. Inspired by a matrix with a symmetric structure such as J_{2^n} has a simple decomposition, we can manually find some particular structures like chains or circles in a graph and first decompose the graph to a linear combination of them. In Fig. 2, we show an example. An eight nodes graph is firstly decomposed into three parts. While other subgraphs with one edge can be handled as before, the first subgraph with a chain from node 0 to node 7 can be decomposed iteratively. A $2^n\times 2^n$ matrix of this kind can be viewed as I tensored by

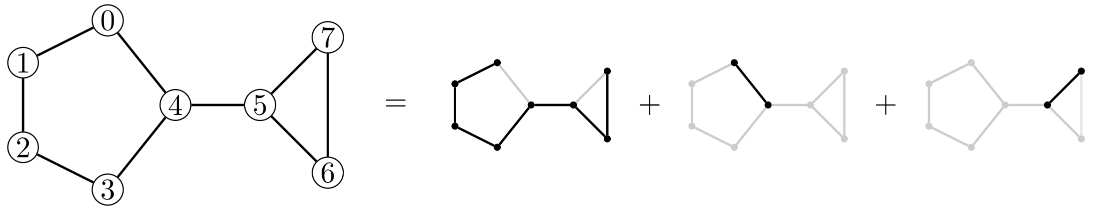

$$\begin{bmatrix}0&1&0&0&1&0&0&0\\1&0&1&0&0&0&0&0\\0&1&0&1&0&0&0&0\\0&0&1&0&1&0&0&0\\1&0&0&1&0&1&0&0\\0&0&0&0&1&0&1&1\\0&0&0&0&0&1&0&1\\0&0&0&0&0&1&1&0\end{bmatrix} = \begin{bmatrix}0&1&0&0&0&0&0&0\\1&0&1&0&0&0&0&0\\0&1&0&1&0&0&0&0\\0&0&1&0&1&0&0&0\\0&0&0&1&0&1&0&0\\0&0&0&0&1&0&1&0\\0&0&0&0&0&1&0&1\\0&0&0&0&0&0&1&0\end{bmatrix} + \begin{bmatrix}0&0&0&0&1&0&0&0\\0&0&0&0&0&0&0&0\\0&0&0&0&0&0&0&0\\0&0&0&0&0&0&0&0\\1&0&0&0&0&0&0&0\\0&0&0&0&0&0&0&0\\0&0&0&0&0&0&0&0\\0&0&0&0&0&0&0&0\end{bmatrix} + \begin{bmatrix}0&0&0&0&0&0&0&0\\0&0&0&0&0&0&0&0\\0&0&0&0&0&0&0&0\\0&0&0&0&0&0&0&0\\0&0&0&0&0&0&0&0\\0&0&0&0&0&0&0&1\\0&0&0&0&0&0&0&0\\0&0&0&0&0&1&0&0\end{bmatrix}$$

$$\begin{aligned} = \; & I \otimes I \otimes X + 1/2 \cdot \mathrm{CNOT}(2,3)(I \otimes X \otimes (I+Z))\mathrm{CNOT}(2,3) \\ & + 1/4 \cdot \mathrm{CNOT}(1,3)\mathrm{CNOT}(1,2)(X \otimes (I-Z) \otimes (I-Z))\mathrm{CNOT}(1,2)\mathrm{CNOT}(1,3) \\ & + 1/4 \cdot X \otimes (I+Z) \otimes (I+Z) + 1/4 \cdot (I-Z) \otimes X \otimes (I-Z) \end{aligned}$$

Supplementary Figure 2: **An example for heuristic decomposition.** An eight-node graph is divided into three parts with their adjacency matrices painted green, brown and blue respectively. Their decompositions are painted the same color as the corresponding matrices.

$2^{n-1} \times 2^{n-1}$ matrix of the same kind plus one edge. So, the adjacency matrix of a chain that runs through all 2^n nodes in the graph can be decomposed to n groups of Pauli product. This does actually reduce the measurement complexity. Someone may argue that we remove the complexity from algorithms to our manual discovery, but all we find here are fragment information that can be found intuitively. Our algorithm will combine those fragment information to build the quantum node embedding, which captures all nodes' relations.

5. EXPERIMENTAL SETTINGS AND DATA

In Fig.3, we list the information about nuclear spins in our experimental sample. The Hamiltonian for the liquid NMR system can be regarded as $\mathcal{H} = \sum_i \pi\nu_i\sigma_z^i + \sum_{i<j} \frac{\pi}{2} J_{ij}\sigma_z^i\sigma_z^j$. In Fig. 3, the ν_i, called the Larmor frequency, is located by $C_i - C_i$ coordinates. Furthermore, the J_{ij}, called the J-coupling strength between spins, is located by $C_i - C_j$ coordinates.

Unit: Hz

C_1	-1706.37			
C_2	41.64	-14541.69		
C_3	1.45	69.69	-12338.34	
C_4	7.04	1.15	72.34	-16746.77
	C_1	C_2	C_3	C_4

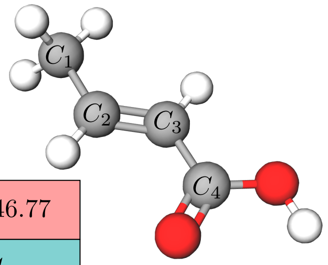

Supplementary Figure 3: **Infomation about carbon-13 nuclear spins in trans-crotonic acid.** The Larmor frequency is given with respect to the reference frequency of 100.613MHz on our 400MHz spectrometer.

In Table. 1-5, we list the experimental data after processing in each iteration step. We can get the expectations of five Pauli product operators for different input parameters by function fitting on their NMR spectrum. Then we can

calculate $\langle A \rangle$, $\langle J_4 \rangle$ and $\mathcal{L}$ based on those items.

Supplementary Table 1: Experimental Data at Step-1 (from t=0 to t=1)

Parameter	$\langle IIIX \rangle$	$\langle IZIX \rangle$	$\langle IXII \rangle$	$\langle IXIZ \rangle$	$\langle IXIX \rangle$	$\langle A \rangle$	$\langle J_4 \rangle$	$\mathcal{L}$
$\boldsymbol{\theta}$	0.0509	-0.8913	0.0538	-0.0616	-0.0305	-0.4547	1.0741	-1.2191
$\boldsymbol{\theta}+\Delta\boldsymbol{\theta_1}$	0.0423	-0.8625	0.0176	-0.1912	0.0012	-0.4958	1.0610	-1.2742
$\boldsymbol{\theta}-\Delta\boldsymbol{\theta_1}$	-0.0012	-0.8819	-0.0034	0.0517	0.0067	-0.4108	1.0020	-1.1172
$\boldsymbol{\theta}+\Delta\boldsymbol{\theta_2}$	0.0500	-0.8655	-0.1033	-0.0405	0.0137	-0.4660	0.9604	-1.1792
$\boldsymbol{\theta}-\Delta\boldsymbol{\theta_2}$	-0.0178	-0.8474	0.1352	-0.0165	-0.0065	-0.3797	1.1109	-1.1251
$\boldsymbol{\theta}+\Delta\boldsymbol{\theta_3}$	-0.0133	-0.8355	-0.0009	-0.1474	0.0428	-0.4558	1.0286	-1.1979
$\boldsymbol{\theta}-\Delta\boldsymbol{\theta_3}$	-0.0033	-0.8467	-0.0511	0.1004	-0.0369	-0.4373	0.9087	-1.1102
$\boldsymbol{\theta}+\Delta\boldsymbol{\theta_4}$	-0.0086	-0.8399	0.0419	-0.0385	-0.1488	-0.5715	0.8844	-1.2994
$\boldsymbol{\theta}-\Delta\boldsymbol{\theta_4}$	0.0143	-0.8738	0.0259	-0.0160	0.1280	-0.2968	1.1682	-1.0293

Supplementary Table 2: Experimental Data at Step-2 (from t=1 to t=2)

Parameter	$\langle IIIX \rangle$	$\langle IZIX \rangle$	$\langle IXII \rangle$	$\langle IXIZ \rangle$	$\langle IXIX \rangle$	$\langle A \rangle$	$\langle J_4 \rangle$	$\mathcal{L}$
$\boldsymbol{\theta}$	0.0381	-0.7843	-0.0157	0.2498	0.3097	0.0536	1.3321	-0.5856
$\boldsymbol{\theta}+\Delta\boldsymbol{\theta_1}$	0.0187	-0.6699	0.1059	0.1286	0.4039	0.1956	1.5286	-0.4709
$\boldsymbol{\theta}-\Delta\boldsymbol{\theta_1}$	0.0056	-0.7488	0.0598	0.5229	0.3107	0.2305	1.3762	-0.3423
$\boldsymbol{\theta}+\Delta\boldsymbol{\theta_2}$	-0.0023	-0.7690	-0.0541	0.3165	0.3029	0.0485	1.2465	-0.5505
$\boldsymbol{\theta}-\Delta\boldsymbol{\theta_2}$	0.0319	-0.7718	0.1316	0.3320	0.2642	0.1261	1.4277	-0.5247
$\boldsymbol{\theta}+\Delta\boldsymbol{\theta_3}$	0.0494	-0.7435	0.0758	0.1406	0.4080	0.1692	1.5332	-0.5128
$\boldsymbol{\theta}-\Delta\boldsymbol{\theta_3}$	0.0521	-0.7805	0.0581	0.5326	0.2929	0.2240	1.4031	-0.3655
$\boldsymbol{\theta}+\Delta\boldsymbol{\theta_4}$	0.0113	-0.8776	0.1136	0.3568	0.1298	-0.0682	1.2546	-0.7296
$\boldsymbol{\theta}-\Delta\boldsymbol{\theta_4}$	0.0871	-0.7108	0.0752	0.3971	0.3539	0.2783	1.5163	-0.3408

Supplementary Table 3: Experimental Data at Step-3 (from t=2 to t=3)

Parameter	$\langle IIIX \rangle$	$\langle IZIX \rangle$	$\langle IXII \rangle$	$\langle IXIZ \rangle$	$\langle IXIX \rangle$	$\langle A \rangle$	$\langle J_4 \rangle$	$\mathcal{L}$
$\boldsymbol{\theta}$	0.1695	-0.4549	0.0986	0.7604	0.4159	0.7026	1.6839	0.2120
$\boldsymbol{\theta}+\Delta\boldsymbol{\theta_1}$	0.1219	-0.4065	0.0856	0.5699	0.6198	0.8052	1.8273	0.2942
$\boldsymbol{\theta}-\Delta\boldsymbol{\theta_1}$	0.1493	-0.4523	0.0326	0.8156	0.3786	0.6512	1.5605	0.1966
$\boldsymbol{\theta}+\Delta\boldsymbol{\theta_2}$	-0.0521	-0.4073	-0.0803	0.7175	0.4802	0.5691	1.3478	0.1797
$\boldsymbol{\theta}-\Delta\boldsymbol{\theta_2}$	0.2454	-0.4501	0.1445	0.7574	0.4136	0.7622	1.8035	0.2415
$\boldsymbol{\theta}+\Delta\boldsymbol{\theta_3}$	0.1248	-0.4749	0.0944	0.7009	0.5457	0.7683	1.7649	0.2699
$\boldsymbol{\theta}-\Delta\boldsymbol{\theta_3}$	0.1133	-0.4519	0.0938	0.8176	0.4218	0.7081	1.6288	0.2477
$\boldsymbol{\theta}+\Delta\boldsymbol{\theta_4}$	0.1338	-0.5865	0.0611	0.7131	0.4319	0.5926	1.6267	0.0756
$\boldsymbol{\theta}-\Delta\boldsymbol{\theta_4}$	0.1049	-0.3626	0.0724	0.7888	0.5197	0.8215	1.6971	0.3837

Supplementary Table 4: Experimental Data at Step-4 (from t=3 to t=4)

Parameter	$\langle IIIX\rangle$	$\langle IZIX\rangle$	$\langle IXII\rangle$	$\langle IXIZ\rangle$	$\langle IXIX\rangle$	$\langle A\rangle$	$\langle J_4\rangle$	$\mathcal{L}$
$\boldsymbol{\theta}$	0.2589	-0.0190	0.1374	0.5683	0.6997	1.1726	2.0961	0.7108
$\boldsymbol{\theta}+\Delta\boldsymbol{\theta_1}$	0.2126	0.0204	0.1376	0.5446	0.7405	1.1980	2.0906	0.7517
$\boldsymbol{\theta}-\Delta\boldsymbol{\theta_1}$	0.1727	-0.0240	0.1477	0.7232	0.5414	1.0512	1.8618	0.6459
$\boldsymbol{\theta}+\Delta\boldsymbol{\theta_2}$	0.0754	-0.0786	0.0527	0.5154	0.6420	0.9244	1.7701	0.5016
$\boldsymbol{\theta}-\Delta\boldsymbol{\theta_2}$	0.3013	-0.0179	0.2552	0.5007	0.6537	1.1733	2.2102	0.6549
$\boldsymbol{\theta}+\Delta\boldsymbol{\theta_3}$	0.2466	-0.0626	0.1875	0.4527	0.7276	1.1397	2.1617	0.6287
$\boldsymbol{\theta}-\Delta\boldsymbol{\theta_3}$	0.2496	-0.0307	0.2166	0.6064	0.5407	1.0616	2.0068	0.5890
$\boldsymbol{\theta}+\Delta\boldsymbol{\theta_4}$	0.2309	-0.2084	0.1902	0.5314	0.6632	1.0352	2.0843	0.5107
$\boldsymbol{\theta}-\Delta\boldsymbol{\theta_4}$	0.2957	0.1238	0.3133	0.4764	0.6853	1.2899	2.2944	0.7877

Supplementary Table 5: Experimental Data at Step-5 (from t=4 to t=5)

Parameter	$\langle IIIX\rangle$	$\langle IZIX\rangle$	$\langle IXII\rangle$	$\langle IXIZ\rangle$	$\langle IXIX\rangle$	$\langle A\rangle$	$\langle J_4\rangle$	$\mathcal{L}$
$\boldsymbol{\theta}$	0.4174	0.3248	0.4117	0.2609	0.7426	1.4501	2.5718	0.8892
$\boldsymbol{\theta}+\Delta\boldsymbol{\theta_1}$	0.4098	0.2991	0.4241	0.2381	0.7916	1.4772	2.6256	0.9030
$\boldsymbol{\theta}-\Delta\boldsymbol{\theta_1}$	0.3782	0.3017	0.3469	0.4990	0.6707	1.4336	2.3958	0.9525
$\boldsymbol{\theta}+\Delta\boldsymbol{\theta_2}$	0.2127	0.3923	0.2438	0.3880	0.6932	1.3117	2.1497	0.8926
$\boldsymbol{\theta}-\Delta\boldsymbol{\theta_2}$	0.4817	0.3291	0.5251	0.2576	0.6839	1.4807	2.6908	0.8757
$\boldsymbol{\theta}+\Delta\boldsymbol{\theta_3}$	0.3485	0.3214	0.3912	0.2457	0.7627	1.4160	2.5023	0.8729
$\boldsymbol{\theta}-\Delta\boldsymbol{\theta_3}$	0.4304	0.2786	0.3996	0.4805	0.6163	1.4108	2.4463	0.8931
$\boldsymbol{\theta}+\Delta\boldsymbol{\theta_4}$	0.3451	0.2475	0.3529	0.4527	0.6738	1.3729	2.3717	0.8734
$\boldsymbol{\theta}-\Delta\boldsymbol{\theta_4}$	0.2835	0.4663	0.3448	0.4376	0.5932	1.3593	2.2215	0.9282

[1] A. W. Harrow, A. Hassidim, and S. Lloyd, Quantum algorithm for linear systems of equations, Physical review letters **103**, 150502 (2009).
[2] A. Y. Kitaev, Quantum measurements and the abelian stabilizer problem, arXiv preprint quant-ph/9511026 (1995).
[3] D. Aharonov, V. Jones, and Z. Landau, A polynomial quantum algorithm for approximating the jones polynomial, Algorithmica **55**, 395 (2009).
[4] M. Motta, C. Sun, A. T. Tan, M. J. O'Rourke, E. Ye, A. J. Minnich, F. G. Brandao, and G. K. Chan, Determining eigenstates and thermal states on a quantum computer using quantum imaginary time evolution, Nature Physics **16**, 205 (2020).
